# Extended-spectrum ß-lactamase-producing *Escherichia coli* among humans, chickens and poultry environments in Abuja, Nigeria

**DOI:** 10.1186/s42522-020-00014-7

**Published:** 2020-05-27

**Authors:** Mabel Kamweli Aworh, Jacob Kwaga, Emmanuel Okolocha, Lyndy Harden, Dawn Hull, Rene S. Hendriksen, Siddhartha Thakur

**Affiliations:** 1 Department of Veterinary and Pest Control Services, Federal Ministry of Agriculture and Rural Development, Abuja, Nigeria; 2 Nigeria Field Epidemiology and Laboratory Training Programme, Abuja, Nigeria; 3 Department of Veterinary Public Health and Preventive Medicine, Ahmadu Bello University, Zaria, Nigeria; 4 Department of Population Health and Pathobiology, College of Veterinary Medicine, North Carolina State University, Raleigh, North Carolina USA; 5 WHO, FAO, EU Reference Laboratory for Antimicrobial Resistance, Technical University of Denmark, National Food Institute, Kgs. Lyngby, Denmark

**Keywords:** *Escherichia coli*, Antimicrobial resistance, Chicken, Extended-spectrum β-lactamases (ESBL), Nigeria

## Abstract

**Background:**

Globally, chicken is known to be a reservoir for the spread of antimicrobial resistance genes to humans. In Nigeria, antimicrobial drugs are readily accessible for use in poultry production, either for preventive or therapeutic purposes. Extended-spectrum beta-lactamase-producing *Escherichia coli* (ESBL-EC) are transmissible to humans because of their zoonotic potentials. People working very closely with chickens either on farms or markets are at greater risk. The aim of this study was to investigate the prevalence and zoonotic transmission of ESBL-EC among poultry-workers, chickens, and poultry environments in Abuja, Nigeria.

**Methods:**

We conducted a cross-sectional study among workers, chickens and poultry environment in selected farms/chicken markets in Abuja. Stool, faecal, and environmental samples were collected from apparently healthy workers, chickens, and farm/market environments from December 2018 to April 2019. Data were collected electronically using an open data kit (ODK) installed on a Smartphone. Antimicrobial resistance was determined using broth micro-dilution methods against a panel of 14 antimicrobial agents. We carried out the phenotypic and genotypic characterization of the isolates. Data were analyzed by computing frequencies, proportions and spearman’s correlation (ρ).

**Results:**

Of 429 samples, 26.8% (*n* = 115) were positive for *Escherichia coli (E. coli)*. Of the 115 *E. coli* isolates, 32.2% (*n* = 37) were confirmed ESBL producers by phenotypic characterization. Prevalence of ESBL-EC was highest among both poultry-workers (37.8%; *n* = 14) and chickens (37.8%; n = 14) followed by the environment (24.3%; *n* = 9). Both human and chicken isolates showed similar patterns of multidrug resistance to tested antimicrobials with a positive correlation (ρ = 0.91). Among ESBL producers, we observed the dissemination of *bla*CTX-M (10.8%; *n* = 4) genes. The coexistence of *bla*CTX-M-15 and *bla*TEM-1 genes was observed in 8.1% (*n* = 3) of the isolates, out of which (66.7%; *n* = 2) were chicken isolates from the farm, while a single human isolate was from the chicken market.

**Conclusions:**

ESBL-EC isolates were prevalent amongst apparently healthy individuals, chickens and the poultry farm/market environment in Abuja. It is important to educate healthcare workers that people in proximity with poultry are a high-risk group for faecal carriage of ESBL-EC, hence pose a higher risk to the general population for the spread of antimicrobial resistance.

## Background

Antimicrobial resistance (AMR) in recent times has been a topical issue and gained global attention owing to the emergence of multi-drug (MDR) resistant organisms such as antimicrobial-resistant *Escherichia coli* [1–3]. Drug-resistant infections in humans and food animals is increasingly a global public health issue requiring measures worldwide [2]. The World Health Organization (WHO) has stated that AMR is a bigger crisis than HIV-AIDS [3]. In 2016, official studies conducted in the UK stated that AMR accounted for about 700,000 deaths annually. It has also been estimated that by 2050 the number of deaths attributable to AMR will increase to 10 million annually if not tackled now, with 40% of these deaths occurring in Africa, second only to Asia [3].

AMR can lead to more deaths since available antimicrobials are no longer effective for the treatment of common infections in humans and animals [4]. It is characterized by the spread of AMR genes and treatment is very expensive. In the animal population, AMR is facilitated by several factors such as inappropriate medication/ route of administration, non-observance of drug withdrawal periods, poor biosecurity measures and poor surveillance amongst others [5]. According to the WHO, increased threat of antibiotic resistance is a direct result of overuse and misuse of antibiotics in animals and humans [6]. The “Path of Resistance” begins by administering antimicrobials to food-animals such as chickens to keep them healthy. These antimicrobials protect the chickens against known bacterial infections. The acquired resistance of the bacteria, however, resists the antimicrobials making them ineffective. Humans become infected by resistant bacteria via various sources such as contact through the food chain, contaminated environment, occupational exposure [6, 7].

Extended-spectrum beta-lactamase-producing *E. coli* (ESBL–EC), which are zoonotic in nature, is one of the commonest resistant pathogens responsible for human infections. Our recently published work showed that occupational exposure for over 10 years to poultry on farms and in live bird markets was a risk factor for acquiring multidrug-resistance (MDR) *E. coli* [7]. It has been documented that drug resistance among *E. coli* isolates has increased globally mainly as a result of the high prevalence of ESBL producing bacteria [8, 9]. This high prevalence of ESBL-EC has resulted from growing reservoirs in food animals such as chickens and the use of antimicrobials [8, 9]. Studies have shown that ESBL genes which were previously found on chromosomes, but now carried on plasmids are derivatives of plasmid-mediated β-lactamases like *bla*TEM as well as environmentally derived types like *bla*CTX-M [9, 10].

It has been documented that ESBL-EC are resistant to several antibiotics especially penicillins and cephalosporins but remain susceptible to cephamycins and carbapenems [11]. Animal food sources such as chickens have been reported as potential reservoirs for the spread of ESBL-EC to humans in close proximity or via the food chain [9, 12, 13]. The mechanism of spread of antibiotic resistance from animals to humans and vice versa remains controversial. Studies have reported that *E. coli* which can be isolated from water, food and farm animals is associated with antimicrobial resistance [14].

A systematic review of all AMR studies done in Nigeria as at 2017 showed that these studies are either in human and animal populations or focused on animals and the food production environment [7, 15]. However, there is paucity of data on ESBL-EC in humans, animals and the food production environments. This is one of the gaps identified in 2017 when Nigeria conducted a situation analysis on AMR in humans, food animals and the environment in response to the 68th World Health Assembly resolution 68.7 [7, 16].

We hypothesized that chickens harbouring ESBL-EC can become potential sources of transmission of resistant genes to humans exposed to chickens based on their occupation as well as to the chicken market or farm environments. To better understand the association between human ESBL-EC isolates and the potential poultry/environmental sources, we investigated the occurrence of ESBL-EC among humans handling chickens, the chickens themselves and selected poultry farms/market environments in Abuja, Nigeria using disc diffusion and whole-genome sequencing. This will generate baseline data for the implementation of the AMR National Action Plan in Nigeria using a One Health approach.

## Methods

### Study design and sample collection

**Fig. 1:**
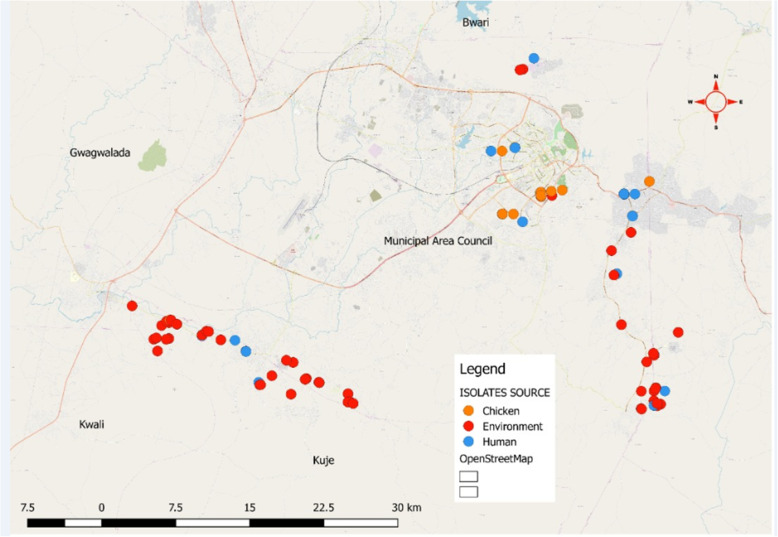
Map of Abuja showing the distribution of ESBL-EC human, chicken and environmental isolates from farms and chicken markets. This map highlights five out of six area councils in Abuja, Federal Capital Territory namely: Bwari, Kwali, Kuje, Gwagwalada and Municipal area councils. Our study was done at two area councils (Kuje and Municipal area councils) with the highest volume of poultry production in Abuja. Each dot represents an ESBL-EC isolate obtained either from humans, chickens or the environment (Source of the Map is the Federal Ministry of Agriculture and Rural Development, Abuja, Nigeria)

This cross-sectional study was conducted in Abuja, North Central Nigeria (Fig. 1). Fifty-two commercial poultry farms and eight live bird markets willingly participated in this study. Sampling took place from December 2018 to April 2019. We collected freshly passed stool samples from randomly selected apparently healthy consenting participants who either worked in poultry farms or chicken markets. The stool samples were collected using sterile stool containers. Fresh faecal samples from chickens that had not contacted the soil were randomly collected using a sterile spoon and stool container. Environmental samples such as litter and water samples were randomly collected from different locations on the poultry farms and live bird markets. We randomly collected 30 g of litter samples from 12 different spots in the poultry houses and 100 mL of water from each study site using sterile containers. All samples were transported in cool boxes to the National Reference Laboratory, Nigeria Centre for Disease Control Gaduwa, Abuja and processed within 3 hours of sample collection for the presence of *E. coli* as previously described [7]*.* A total of 429 samples were collected for this study, comprising 122 human stool samples, 111 chicken samples, and 196 environmental samples. Some aspects of the study in humans focusing on the risk factors for acquiring multidrug-resistant *E. coli* has already been published in recent times [7].

### Microbiological analysis

#### Isolation and identification of *E. coli* isolates

Briefly, about one gram of human stool sample, one gram of poultry faeces and 30 g of litter samples were inoculated respectively in enrichment broth (buffered peptone water) in a 1:10 sample to broth ratio and incubated at 37 °C for 24 h. Subsequently, a 10ul loop-full of overnight culture from enrichment broth was plated onto MacConkey lactose agar and incubated at 37 °C for 24 h. Suspected *E. coli* colonies, usually pink to red were picked and further streaked on Eosin Methylene Blue (EMB) agar. Colonies suggestive of *E. coli* were then sub-cultured onto Tryptic Soy Agar (TSA) plates and incubated for 24 h at 37 °C under aerobic conditions for the isolation of pure cultures as previously described [7]. All the *E. coli* isolates were further tested for indole, methyl red, Voges–Proskauer, and citrate utilization biochemical tests [7]. Isolates that were presumptive for *E. coli* in the screening tests were later subjected to further testing using the commercially available biochemical test strip, Microbact GNB 24E (Oxoid, UK), for confirmation according to the Manufacturer’s instructions.

#### Isolation of *E. coli* from water samples

For the isolation of *E. coli* from poultry farm/live bird market water samples; the Membrane filtration technique was used for the isolation and the identification of *E. coli* from water samples. Single sterile 0.45 μm pores filter disks were placed in a filtration unit to filter each 100 ml of the water sample. The filter membranes were then placed on EMB agar plates and incubated at 37 °C for 24 h. All the *E. coli* isolates were further tested as stated above.

### Antimicrobial susceptibility testing

The minimum inhibitory concentrations (MIC) of ESBL-EC isolates were determined by broth microdilution assay methods using the Gram-negative Sensititre™ (CMV3AGNF) plate (Trek Diagnostic Systems, OH) against a panel of 14 antimicrobial agents. Of these, five were β-lactam antimicrobials comprising ampicillin, amoxicillin/clavulanic acid, ceftriaxone, ceftiofur, and cefoxitin while the rest were non-β-lactam antimicrobials (streptomycin, gentamicin, azithromycin, ciprofloxacin, chloramphenicol, trimethoprim/sulfamethoxazole, tetracycline, sulfisoxazole, and nalidixic acid. Briefly, three distinct colonies were picked from the overnight culture on TSA plates and suspended into 4 ml of sterile deionized water. Next, this was adjusted to a 0.5 McFarland standard after which 10 μl of suspension was mixed with Mueller-Hinton broth. Next, 50 μl of the suspension was inoculated to each well of a Sensititre™ plate using the Sensititre™ auto-inoculator (Trek Diagnostic Systems, OH) and incubated at 37 °C for 24 h. Plates were read using Sensititre™ ARIS automated system which interprets isolates based on the MIC as susceptible, intermediate or resistant using the guidelines of Clinical and Laboratory Standards Institute (CLSI) M100 28th Edition [17]. *E. coli* ATCC25922 was used for internal quality control and categorized ESBL-EC isolates with intermediate MIC levels as resistant [17]. We defined multidrug resistance (MDR) as resistance to three or more classes of antimicrobials.

### Phenotypic and genotypic detection of ESBLs

#### Detection of ESBL phenotype by disk diffusion test

All the *E. coli* isolates were screened for the production of extended-spectrum beta-lactamase (ESBLs) by using the disk diffusion test as described by CLSI guidelines (M100 28th Edition) [17]. From the pure cultures of bacteria grown overnight on TSA plate supplemented with sheep blood, a suspension matching 0.5 McFarland standard (1.5 × 108 CFU/ml) was prepared in normal saline. A sterile cotton swab was used to spread the bacteria on Mueller Hinton agar in order to obtain a lawn culture. After allowing the plate to dry, disks of ceftazidime (30 μg) (CAZ), ceftazidime + clavulanic acid (30/10 μg) (CAC), cefotaxime (30 μg) (CTX), cefotaxime + clavulanic acid (30/10 μg) (CEC) were placed on the surface and the plates were incubated in ambient air at 37 °C for 16–18 h. Following growth, the diameter of the zones around the disks was measured and recorded. An increase in the zone diameter by ≥5 mm around the disks containing cephalosporin with clavulanic over the disks containing cephalosporin alone indicated ESBL production according to CLSI guidelines (M100 28th Edition). *E. coli* ATCC 25922 and *K. pneumoniae* ATCC 700603 were used as negative and positive controls respectively [17].

#### Whole genome sequencing (WGS) of *E. coli* isolates

All *E. coli* isolates from humans, poultry and environmental samples were subjected to whole-genome sequencing. Briefly, all *E. coli* isolates (*n* = 110) were cultured overnight at 37 °C on Luria-Bertani (LB) agar for 24 h. DNA was extracted using Whole Genome DNA Isolation for Gram-Negative Bacteria protocol (Lucigen MasterPure™ Gram Positive DNA Purification Kit) following the manufacturer’s instructions. Next, DNA concentrations were quantified using the Qubit 4.0 Fluorometer assay kit (Thermo Fisher Scientific, MA). After DNA quantification, libraries for each *E. coli* isolates were prepared for WGS using a Nextera XT kit. Briefly, 0.3 ng/μL of DNA from each isolate was processed using a Nextera XT DNA Sample Prep Kit (Illumina Inc., San Diego, CA), pooled together, and sequenced on an Illumina Miseq platform using a 2 × 250 paired-end approach (Illumina Inc., San Diego, CA). Raw sequencing reads were demultiplexed and converted to fastq files using CLC Genomics workbench version 9.4 (Qiagen bioinformatics, Valencia, CA). The DNA sequences for each isolate were transferred to the National Center for Biotechnology (NCBI) database after which each isolate was given an accession number. *In silico* prediction of antimicrobial resistance was conducted by comparing the DNA sequence for each isolate against several genetic analysis databases such as ResFinder (version 3.2, database date: 2019-07-02, cge.cbs.dtu.dk), Antibiotic Resistance Gene-ANNOTation (ARG-ANNOT), ABRICATE and the Comprehensive Antibiotic Resistance Database (CARD) to identify resistance genes [18]. For each isolate, we used between 90 and 100% identity to match individual genes to an annotated resistance gene. However, the genes included in the final profile were decided using the output from ResFinder version 3.2 [19].

### Multi-locus sequence typing (MLST) of MDR *E. coli* isolates

We performed *in silico* MLST-analyses using previously described schemes by Achtman [20] which considered allelic variation amongst seven housekeeping genes (*adk, fumC, gyrB, icd, mdh, purA, and recA)* to assign sequence types (STs). WGS data were used to generate the *E. coli* MLST assignment for each isolate that perfectly matched the alleles in the MLST database [20]. Isolates with 100% match against known MLST alleles were assigned STs however those without perfect matches were usually identified as non-conclusive or unknown. Some isolates which were matched with MLST alleles of unknown ST in the MLST database were assigned as potential new type [21].

### Data collection and analyses

Data were collected electronically using open data kit (ODK) installed on a smartphone. Data were analyzed by computing frequencies, proportions and spearman’s correlation (ρ). Out of 110 *E. coli* isolates sequenced from our study, the accession numbers for 108 paired-end reads have been deposited by the Thakur Molecular Epidemiology Laboratory, NC State University (GenomeTrakr Project) in the National Center for Biotechnology Information under the Bio project ID number PRJNA293225. The remaining two isolates have accession obtained from the DNA Data Bank of Japan (DDBJ) [22, 23]. The additional data file for this study contains a list of accession numbers for individual Sequence Read Archive (SRA) for the ESBL-EC isolates.

## Results

### Prevalence of *E. coli* in humans, chickens and poultry farm/market environment

Overall, 429 samples comprising human stool (*n* = 122), chicken faeces (*n* = 111), litter (*n* = 131) and water samples (*n* = 65) were collected from 52 poultry farms and 8 chicken markets in Kuje and Municipal Area councils of Abuja, Nigeria (Fig. 1). The sample size from each farm or market varied depending on the actual size in terms of the number of chickens reared or sold and the willingness of the owners to participate in the study.

**Fig. 2:**
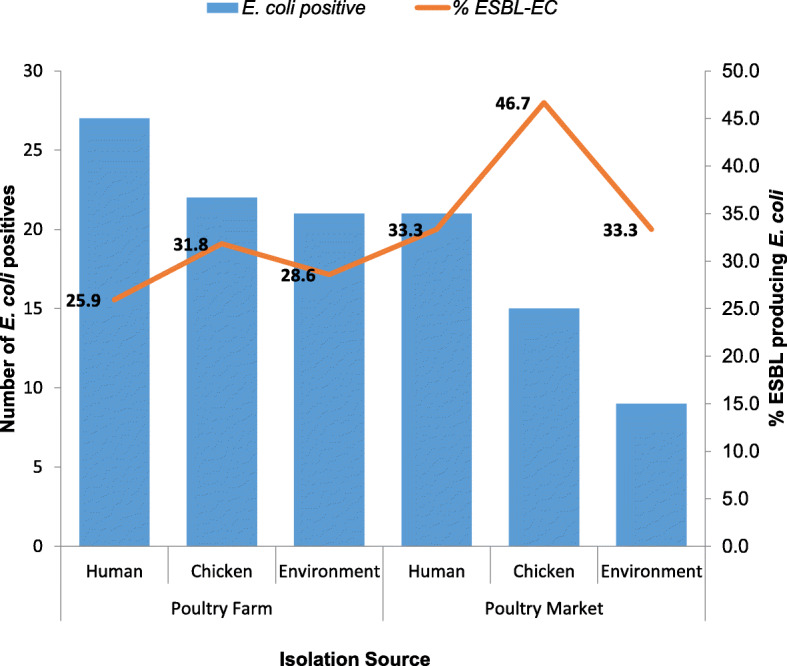
Proportion of ESBL-EC among positive *E. coli* isolates from humans, chickens, and poultry environment in Abuja, Nigeria, 2019. The number of *E. coli* positive isolates are plotted as bars on the primary axis while the proportion of these positive isolates which are ESBL-EC in percentages are plotted as a line graph on the secondary axis. The various data points on the line graph are also displayed on the chart

The overall prevalence of *E. coli* from all sources was 26.8% (*n* = 115) out of which 61% (*n* = 70) were obtained from poultry farms and 39.1% (*n* = 45) from chicken markets. Out of a total of 70, *E. coli* isolates from the poultry farms, 38.6% (*n* = 27) were of human origin; 31.4% (*n* = 22) of poultry origin, while 30% (*n* = 21) were from the poultry farm environment (litter and water samples). Forty-five *E. coli* isolates were obtained from the chicken markets out of which 46.7% (n = 21) were human isolates, 33.3% (*n* = 15) were chicken isolates and 20% (*n* = 9) were from the chicken market environment (Fig. 2).

### Detection of ESBL-EC isolates

**Fig. 3:**
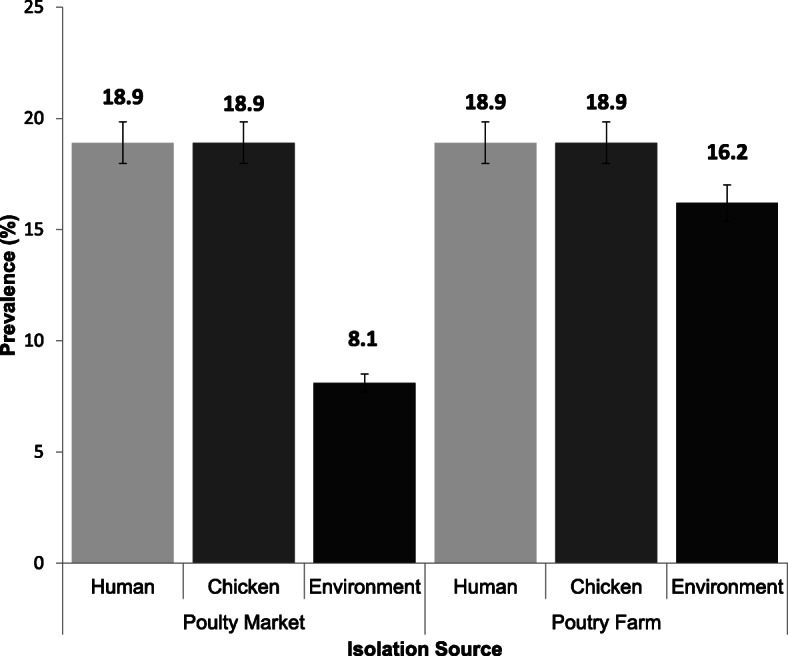
Prevalence of ESBL-EC isolated from humans, chickens and poultry environment, Abuja, Nigeria, 2019. Bars represent the proportion of ESBL-EC isolates from each isolation source with 95% confidence intervals. Error bars represent Standard Error of the mean prevalence. Data were obtained from two sources: poultry farms and poultry markets

Of the 115 *E. coli* isolates, 32.2% (*n* = 37) were confirmed ESBL producers by phenotypic characterization. Prevalence of ESBL-EC was highest among both poultry-workers 37.8% (*n* = 14) and chickens 37.8% (n = 14) followed by the poultry farm/market environment 24.3% (n = 9). Two (22.2%) of the environmental isolates obtained from litter and water sources  were from the same chicken market. More than half (54%) of these isolates were from samples obtained from the poultry farm. The human and chicken isolates from the farm and chicken markets had similar ESBL-EC prevalence; however, the environmental isolates from the farm had doubled the prevalence of that observed in the chicken markets (Fig. 3).

### Antimicrobial susceptibility testing of ESBL-EC isolates

Of 37 ESBL-EC isolates, 94.6% (*n* = 35) were multidrug-resistant (MDR). Out of these, 37.1% (*n* = 13) were human isolates, 37.1% (n = 13) were chicken isolates while 25.7% (*n* = 9) were environmental isolates (Table 1). Overall, the frequency of resistance in ESBL-EC to the antibiotics tested were tetracycline (97.3%), ampicillin (97.3%), sulfamethoxazole + trimethoprim (83.7%), sulfisoxazole (81.1%), Cefoxitin (78.4%), streptomycin (75.7%), gentamicin (45.9%), nalidixic acid (45.9%), azithromycin (35.1%), chloramphenicol (32.4%), and ciprofloxacin (27.0%).

**Table 1:** Antimicrobial resistance profiles of ESBL-EC isolates from Humans, chickens and farm/market environments in Abuja - Nigeria, 2019

Drug Class	Drug	MIC Resistance breakpoint μg/mL	Humans, ***n*** = 14 (%)	Chickens n = 14 (%)	Environment ***n*** = 9 (%)
**Tetracyclines**	Tetracyclines (TET)	≥ 16	14 (100)	13 (92.9)	9 (100)
**Folate Pathway antagonists**	Sulfamethoxazole Trimethoprim (SXT)	≥ 4/76	12 (85.7)	10 (71.4)	9 (100)
	Sulfisoxazole (FIS)	≥512	12 (85.7)	9 (64.3)	9 (100)
**Penicillins**	Ampicillin (AMP)	≥ 32	13 (92.9)	14 (100)	9 (100)
**Quinolones**	Nalidixic acid (NAL)	≥ 32	5 (35.7)	7 (50.00)	5 (55.6)
	Ciprofloxacin (CIP)	≥ 4	3 (21.4)	5 (35.7)	2 (22.2)
**Aminoglycosides**	Streptomycin (STR)	≥ 32	10 (71.4)	10 (71.4)	8 (88.9)
	Gentamicin (GEN)	≥ 16	5 (35.7)	7 (50.00)	5 (55.6)
**Phenicols**	Chloramphenicol (CHL)	≥ 32	3 (21.4)	7 (50.00)	2 (22.2)
**Macrolide Antibiotics**	Azithromycin (AZI)	≥ 32	4 (28.6)	4 (28.6)	5 (55.6)
**B-lactam inhibitors**	Amoxicillin-clavulanate (AMC)	≥ 32/16	1 (7.14)	0 (0)	0 (0)
**Cephem**	Ceftriaxone (CRO)	≥ 4	2 (14.3)	2 (14.3)	0 (0)
	Cefoxitin (FOX)	≥ 32	14 (100)	14 (100)	1 (11.1)
	Ceftiofur (XNL)	≥ 8	2 (14.3)	2 (14.3)	1 (11.1)
**Resistance to 3 or more classes of antibiotics**	MDR	n/a	13 (92.9)	13 (92.9)	9 (100)

### ESBL level genes in humans, chickens and poultry environment

Both human and chicken isolates showed similar patterns of multidrug resistance to tested antimicrobials with a positive correlation (ρ = 0.91). Among ESBL producers, we observed the dissemination of *bla*CTX-M 10.8% (n = 4) genes out of which 50% (*n* = 2) were chicken isolates from different chicken farms while 50% (*n* = 2) were human isolates from the same chicken market. Of the *bla*CTX-M genes detected, 75% (*n* = 3) were of the subtype *bla*CTX-M-15 while 25% (*n* = 1) was of the subtype *bla*CTX-M-65. Both human isolates and one chicken isolate were of the *bla*CTX-M-15 subtype.

### Resistance determinants detected in ESBL-EC isolates

This study identified more than 30 different resistance determinants from 37 ESBL-EC isolates (Table 2). Aminoglycosides accounted for the majority of these resistance determinants with about 10 different variants (*aadA1*, *aadA2*, *aadA5*, *aac(3)-IIa*, *aac(3)-IId*, *aac(3)-Ib*, *aac(6)-Ib-cr*, *aph(3)-Ia*, *aph(3)-Ib*, *aph(6)-Id)* detected. More than half of the ESBL-EC isolates (25) exhibited *aph(3)-Ib* gene which is a metabolic enzyme conferring aminoglycoside resistance. It is important to note that we detected *aac(6)-Ib-cr* gene which is responsible for the reduction in the activity of ciprofloxacin in one ESBL-EC isolate. We also observed the *aac(3)-IId* gene which is responsible for conferring resistance to gentamicin among ten ESBL-EC isolates. Among the aminoglycoside resistance genes in high prevalence was *aph(6)-Id* which is a plasmid-encoded gene. This was followed by β-lactam resistance genes which were of six different types (*bla*TEM-1, *bla*TEM-20, *bla*OXA-1, *bla*OXA-129, *bla*CTX-M-15, *bla*CTX-M-65) out of which blaCTX-M type was classical of the ESBL producing *E. coli*. Resistance to fluoroquinolones, one of the WHO listed critically important antimicrobials, were also detected with five different variants (*qnrB1*, *qnrB19*, *qnrS1*, *qnrS2*, *aac(6)-Ib-cr)* and this is usually associated with mutations in the *gyr*A and *par*C genes. Other resistance determinants that we observed included phenicol resistance (*cmlA1*, *catA1*, *catA2*, *catB3*, *floR)*, rifampicin resistance (ARR-3), sulphonamide resistance (*sul1*, *sul2*, *sul3*), tetracycline resistance (*tet*A, *tet*B) and trimethoprim resistance (*dfrA1*, *dfrA12*, *dfrA14*, *dfrA17, dfrA21).* Our study detected one plasmid-mediated colistin resistance gene (*mcr*-1.1).

**Table 2:** Resistance determinants detected in ESBL-EC isolates from humans, chickens and farm/market environment in Abuja - Nigeria, 2019

Antibiotic class^**a**^	Resistance determinants of ESBL-EC isolates (no of isolates)^**b**^
Aminoglycoside	*aadA1* (6), *aadA2* (4), *aadA5* (3), *aac(3)-IIa* (4), *aac(3)-IId* (11), *aac(3)-Ib* (1), *aac(6)-Ib-cr* (1), *aph(3)-Ia* (6), *aph(3)-Ib* (25), *aph(6)-Id* (22)
β-lactamases	*bla*TEM-1 (33), *bla*TEM-20 (1), *bla*OXA-1 (1), *bla*OXA-129 (1), *bla*CTX-M-15 (3), *bla*CTX-M-65 (1)
Colistin	*mcr-*1.1 (1)
Macrolide	*mdf*A (34), *mph*A (11), *mph*B (1)
Phenicol	*cmlA1* (2), *catA1* (4), *catA2* (1), *catB3* (1), *floR* (6)
Rifampicin	*ARR-3 (1)*
Quinolone	*qnrB1* (1), *qnrB19* (6), *qnrS1* (24), *qnrS2* (2), *aac(6)-Ib-cr* (1)
Sulphonamide	*sul1* (7), *sul2* (30), *sul3* (3)
Tetracycline	*tet*A (31), *tet*B (3)
Trimethoprim	*dfrA1* (3), *dfrA12* (3), *dfrA14* (23), *dfrA17* (4), *dfrA21* (1)

^a^Drugs corresponding to each antibiotic class used in our study are as follows: aminoglycosides, streptomycin, gentamicin; beta-lactams, ampicillin, ceftriaxone, cefotaxime, ceftazidime, ceftiofur, cefoxitin, amoxicillin-clavulanic acid, and cefpodoxime; quinolones, nalidixic acid, and ciprofloxacin; phenicols, chloramphenicol; sulfonamides and trimethoprim, trimethoprim-sulfamethoxazole, Sulfisoxazole; tetracyclines, tetracycline; macrolides, azithromycin.

^b^The number of isolates carrying each resistance determinant are presented in parentheses

### Multi-locus sequence determination of ESBL-EC isolates

**Fig. 4:**
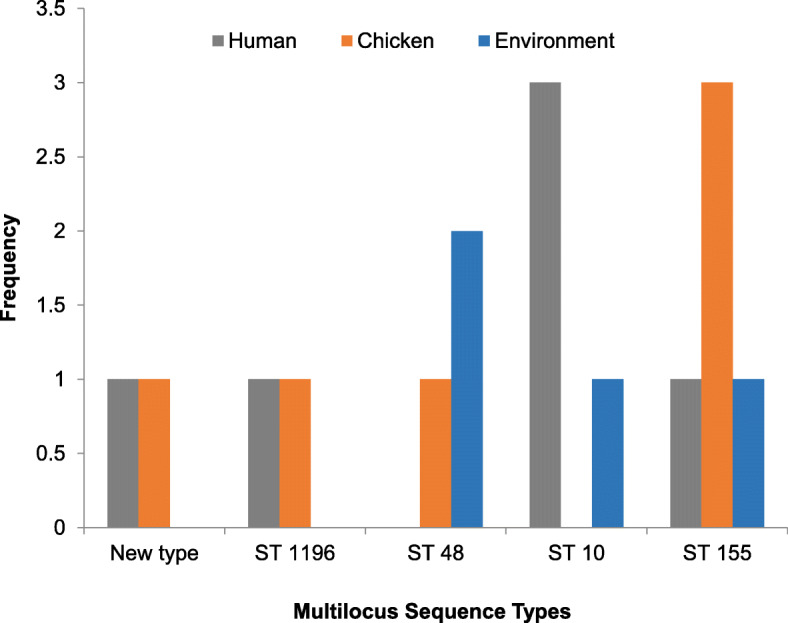
Multilocus Sequence Types for ESBL-EC isolated from humans, chickens and poultry environment, Abuja, Nigeria, 2019. Each bar represents the various ESBL-EC sequence types for isolates obtained from humans, chickens and chicken farm/market environments

The 37 ESBL-EC isolates belonged to 27 different sequence types (ST), out of which one was non-conclusive and two were novel. ST-155 (13.5%; *n* = 5), ST-10 (10.8%; *n* = 4), ST-48 (8.1%; *n* = 3) and ST-1196 (5.4%; *n* = 2) were the most commonly observed STs in silico analysis of ESBL-EC (Fig. 4).

In the ST-10 group, the most represented isolate was from humans handling chickens on the farms and in the chicken market (3/4), while the least common ST in this group was from the chicken market environment (1/4). This was followed by the ST-155 group majority of which were from the chicken market (3/5 from chickens, 1/5 from human and 1/5 from the poultry market environment). The most common ST observed in the poultry farm environment was ST-48 (2/3). The *in silico* β-lactamase analysis of the ESBL-EC showed that the most detected gene was *bla*CTX-M (10.8%). All of the *bla*CTX-M isolates belonged to four different sequence types; being a novel ST, a non-conclusive ST, ST-10, and ST-2179.

## Discussion

Globally, studies have documented that *E. coli* isolated from food-producing animals particularly chickens are usually resistant to **β**-lactam antimicrobial agents [7, 24, 25]. It has also been reported that chickens are an important source of ESBL-EC transmission to humans although this has not been clearly demonstrated [1, 13, 26].

Our study attempted to investigate the prevalence of ESBL-EC among people who handle chickens either on the farm or market as well as in the various environment where the chickens are reared or sold. Our findings showed that antimicrobial-resistant *E. coli,* particularly ESBL-EC, are present in the poultry farm environment, as well as the market environment, where these chickens are sold acting as a reservoir of antimicrobial-resistant bacteria and eventually pose as a health risk to humans working in such environment. This study observed that the prevalence of ESBL-EC was 37.8%, 37.8% and 24.3% in humans, chickens and poultry environment, respectively. Our study prevalence of ESBL-EC in humans, animals, and the environment was higher than what was observed in another similar study [2, 12]. Similar studies conducted elsewhere reported a much higher prevalence of ESBL-EC in chickens and apparently healthy individuals [11, 12]. Our study prevalence of ESBL antimicrobial resistance in human and chicken isolates was similar however, a much lower prevalence was observed in the environmental isolates. A possible explanation may be as a result of easy access to antimicrobials for both human and veterinary use as opposed to the developed economies where these are strictly prescription-only medicines [15, 27, 28].

Tetracycline, sulphonamides and aminoglycosides class of antimicrobials accounted for the majority of the resistance determinants observed in this study. This is similar to the findings of other studies that reported high tetracycline and sulphonamides resistance on poultry farms [12, 15, 27, 28]. The high prevalence of AMR genes belonging to these classes of antimicrobials observed in this study is most likely because these antimicrobials are commonly used in chicken farms in Nigeria either for prophylaxis or therapeutic purposes [15, 27, 28].

Our study showed that among the ESBL-EC isolates, one of the most prevalent resistance genes observed were *tet*A and *tet*B that are responsible for resistance against tetracycline. This finding was not surprising as tetracycline is one of the most abused antibiotics in poultry production in Nigeria [27, 29, 30].

In our study, *bla*CTX-M was the only ESBL gene type observed among human and chicken isolates and this is consistent with findings from other studies [13, 31, 32]. The *bla*CTX-M-15 genes detected in this study were observed in human isolates from the same chicken market however our study cannot clearly demonstrate that this occurred as a result of horizontal gene transfer from one chicken seller to the other. The chicken isolates had two different CTX-M subtypes: CTX-M-15 and CTX-M-65. These chicken isolates where CTX-M genes were observed were also sourced from two different poultry farms. Interestingly, the CTX-M-15 subtype from one chicken isolate was similar to that of the human isolates. It is important to note that chickens sold in the chicken markets in Abuja are usually sourced from various farms, thus providing an enabling environment for the spread of antimicrobial resistance genes.

Although studies have provided some evidence that food animals are critical in the transfer of antimicrobial-resistant *E. coli* to the human population, our study cannot clearly demonstrate that the resistance observed in the apparently healthy humans were from the chickens or the farm/market environment. In contrast to our findings, a study done in Ghana demonstrated that four human isolates and broiler isolates were closely related suggesting a possibility of spread of resistance between the two populations [13]. The possible explanation for the disparity in our findings when compared with the documented evidence by the Ghana study was that the isolates in the Ghana study were obtained from sick children while our source was apparently healthy individuals.

The genetic relatedness of the ESBL-EC isolates in our study was determined using multilocus sequence typing (MLST) of whole-genome sequences. Our findings showed the genetic diversity of ESBL-EC as we observed 27 different sequence types (STs) and this is consistent with findings from other studies [12, 13, 31, 32]. However, the most common STs were ST-10, ST-48 and ST-155. In our study, ST-10 was observed in humans and chicken farm/market environment but not in chickens although previous studies have reported ST-10 as one of the most common in human and chicken populations [13, 32]. Our findings showed that the ESBL-EC strains from the humans, chickens, and chicken market environments had identical ST-155, thus suggesting that a possible transmission between these hosts and the environment may have occurred. This finding demonstrates that the co-colonization of antimicrobial-resistant *E. coli* from a shared source is also possible. There is documentary evidence that *E. coli* ST-155 represents an important zoonotic strain responsible for the transmission of ESBL-EC *t*o humans [33, 34]. It is also important to note that ST-155 has been detected in *E. coli* isolates from water samples [34] and this is in agreement with findings from our study where this ST was detected in isolates from environmental samples. A possible explanation for our findings is that there is a possibility for the circulation of host-adapted lineages of ESBL-EC.

To the best of our knowledge, this study is the first in Nigeria to report plasmid-mediated colistin resistance gene (*mcr*-1.1) in the chicken market environment. The *mcr*-1.1 positive *E. coli* strain was of ST-10 and isolated from poultry water at the chicken market. This finding is rather not surprising as colistin, which is considered an antimicrobial agent of last resort is often used for therapeutic purposes in poultry production in Nigeria [7, 27]. Our results are in agreement with documentary evidence that ST-10 is the most common ST globally carrying the *mcr*-1 gene as well as the dominant ST in both animal and water samples [35]. This finding has highlighted the potential for the *mcr*-1 gene to spread from the poultry farm to the chicken market environment, hence should be considered a potential public health risk especially to the chicken sellers.

## Conclusions

ESBL-EC isolates were prevalent amongst apparently healthy individuals, chickens and the poultry farm/market environment in Abuja. Among ESBL-EC, the highest resistance was observed to tetracycline, sulphonamides, and aminoglycosides which are classes of antimicrobials commonly used on chicken farms for therapeutic purposes in Abuja. Our study detected ESBL genes, *bla*CTX-M in human isolates from the same chicken market however, the chicken isolates with *bla*CTX-M genes were sourced from two different poultry farms. ST155 was the only ST detected in humans, chickens and chicken farm environments in this study. The possibility of the spread of ESBL-EC from other environmental sources on the chicken farm/market to either humans or chickens may have been due to poor biosecurity measures observed on the farms or markets where samples were collected. It is therefore important to educate healthcare workers that people in proximity with poultry are a high-risk group for faecal carriage of ESBL-EC, hence pose a higher risk to the general population for the spread of AMR. It is also important to educate farmers on the need to observe biosecurity measures on chicken farms and markets.

## Supplementary information

**Additional file 1.**

## Data Availability

The datasets used and analyzed during the current study are available from the corresponding author on reasonable request. All data generated or analyzed during this study are also included in this published article [and its supplementary information files].

## Abbreviations

AMR: Antimicrobial resistance
ARG-ANNOT: Antibiotic Resistance Gene-ANNOTation,
CARD: Comprehensive Antibiotic Resistance Database
CLSI: Clinical and Laboratory Standards Institute
DDBJ: DNA Data Bank of Japan
ESBL: Extended-Spectrum Beta-Lactamase
ESBL-EC: Extended-Spectrum Beta-Lactamase producing *E. coli*
LB: Luria-Bertani
MDR: Multi-drug Resistance
MIC: Minimum Inhibitory Concentration
MLST: Multi-locus Sequence Typing
NCBI: National Center for Biotechnology Information
ODK: Open data kit
SRA: Sequence Read Archive
ST: Sequence Type
TSA: Tryptic Soy Agar
WGS: Whole-genome Sequencing
WHO: World Health Organization
